# On the Identity of the White Corpuscles of the Blood, with the Salivary Pus, and Mucous Corpuscles

**Published:** 1870-12

**Authors:** Joseph Richardson


### ARTICLE VIII.

### *On the Identity of the White Corpuscles of the Blood, with the Salivary Pus, and Mucous Corpuscles.*

By Joseph Richardson, M. D.

The nature of the nucleated corpuscles so abundant in the saliva has long been a subject of some uncertainty, although they have probably, as favorite tests for the higher powers, been more frequently examined by microscopists than almost any other constituent of the glandular secretions. Observers seem to have been generally content to accept them simply as useful measures for the capacity of the higher objectives, and passed on without any attempts to solve the mystery of their origin ; Kölliker, indeed, advanced the theory that they were essentially a form of exudation corpuscles, but hitherto, his hypothesis does not appear to have generally accepted by microsopists as a fixed fact.

The following experiments, undertaken to elucidate their constitution, were performed with the large Powell and Lealand's instrument, so long a denizen of the "Microscope Room," in the Pennsylvania Hospital, and no doubt endeared by constant association to many generations of "Residents," as well as to myself. When it was discarded by the Institution I became the purchaser, and after undergoing some repairs, and having adapted it to a 1.25th inch objective (made by Mr. William Wale of Fort Lee, N. J.,) it has accomplished the work below described.

The salivary corpuscles examined under a power of eleven hundred diameters present the appearance of perfect spheres, varying from the 1.1400th to the 1.2500th of an inch in diameter, each having a very transparent but beautifully defined cell-wall of exceeding tenuity, which encloses from one to four almost equally transparent nuclei of a circular or oval form, whose diameters range from 1.3000th to 1.4000th of an inch. These nuclei are situated sometimes centrally, but more commonly near one side of the corpuscle, and the cavity between the margin and the cell-wall is generally filled with from twenty-five to fifty molecules, not more than 1.20000th of an inch in diameter, whose characteristic is that of rapid and constant motion. Some of these molecules seem to be elongated into an oval or hour-glass form, but the activity of their movements renders it difficult to ascertain this with precision. In my observations these corpuscles have appeared to enlarge and become flattened, from the pressure of the glass cover, as the stratum of liquid beneath became thinner from marginal desiccation, so that usually in the course of an hour or so they burst, and discharge about one fourth of their contents, when two, three or more of the molecules swim away, continuing their revolving movements until they pass out of view; the other granules outside and those remaining within the cell become within a very few seconds entirely stationary. If a solution of aniline red, of the strength of one grain to the ounce of distilled water, be allowed to penetrate at the margin of the cover, the nuclei of the salivary corpuscles are readily stained of a bright crimson, and are thus exhibited with beautiful distinctness; the dye appears, however, to exert an immediate influence upon the movement of the molecules, as I have rarely been able to find cells in which they continued to move after the nuclei became at all colored.

In examining some urine, obtained on the 8th of August, 1869, near my late residence, in Western New York, from a patient who complained of severe pain in the kidneys and bladder, I was surprised to find that a deposit, which appeared to the naked eye purulent, was chiefly composed of cells, exactly resembling in form, size, definite cell-wall, contained nuclei, and actively revolving moleclues, the salivary corpuscles with which I had become so familiar; and should have imagined that these preceeded from an accidental adulteration with sputum, had I not been fortunate enough to have ocular demonstration to the contrary when procuring the specimen. I examined the corpuscles repeatedly in the course of the two following days, during which the movements of the molecules continued, but could make nothing else of them except drawings which I carefully preserved.

On consulting the text-books to which I had access, I found that neither Beale, Roberts, Bird, nor Naubauer & Vogel, in their works on the urine, mentioned cells such as those above described, although the editors of the "Micrographic Dictionary," in their description of the salivaay corpuscles, state that they have seen them by myriads in the renal secretion; nevertheless, numerous specimens examined during the following few months, seldom without special scrutiny for similar bodies, afforded none, until in a deposit occuring from urine brought me by a medical friend about December 1st, the corpuscles I had so long been in search of were at last recognized, and on this occasion I was able to exhibit them to several microscopists, among others to my friend, Dr. H. C. Wood, Jr., Professor of Botany, in the University of Pensylvania.

On the 5th of December, I procured a sample of urine from a case of cystitis, which had only been passed a few hours, and on placing it under the field of the 1.25, I found many of the pus-globules exhibiting the amœboid movements described by Dr. Beale, in his late elaborate work on the "Microscope in Practical Medicine," no corpuscles containing moving molecules were visible, but observing that some of the pus-cells having a spherical outline were almost opaque, and only about 1.3000 of an inch in diameter, it occurred to me they were perhaps only contracted by the exosmose of their fluid contents into the surrounding denser medium, and the idea suggested itself to try the effects of diminishing the specific gravity of the urine, by the addition of water. Under this treatment, I found that the cells which had been exhibiting amœboid movements, soon assumed a spherical shape, rapidly enlarged until they reached the diameter of about 1.1700 of an inch, when the contained molecules began to revolve, and ere long took upon themselves the extremely rapid and confused movement which I had twice before seen in cells occurring in urine, and hundreds of times in the salivary corpuscles; the action of aniline solution rendered beautifully distinct, definite nuclei similar to those found in the salivary bodies.

The opportunity of corroborating the interesting and remarkable researches of Dr. Cohnheim, of Berlin, on the identity of the pus, and white blood corpuscles thus obviously presenting itself, I proceeded with the following experiments. Drawing a drop of blood from the tip of my finger upon a "growing slide," I covered with thin glass, and placed it upon the stage of the microscope, after finding a white blood-corpuscle showing well marked granules,

I raised the objective, and arranged a fine filament of thread from the reservoir filled with fresh water to the upper edge of the cover, and a fragment of wet paper to the lower, according to the usual method for securing a constant current beneath the thin glass. On depressing the body of the instrument and bringing the corpuscles again into view, I found it still adhering to the surface of the cover, notwithstanding the torrent of red globules hurrying over the field; and these became paler and less distinct by reason of the diminished density of the serum. The white cell first gradually expanded and displayed its delicate wall with two rounded nuclei, then, after acquiring the magnitude of about one 1700 of an inch, it exhibited the rapid and incessant movement of its contained molecules, and finally, when its diameter reached about one 1400th of an inch, it burst suddenly, discharging a portion of its contents, whose outbreak resembled that of swarm of bees from a hive, and some particles of which, actively revolving as they went, swarm off to the confines of the field. On repeating the observation and allowing some of the aniline solution to flow in with the water after the first few minutes, the nuclei were strongly stained and rendered beautifully distinct, although the movement of the molecules promptly ceased, in this respect as in all the others showing a precise identity with the reactions afforded by the pus and the salivary corpuscles as above described. It should be noted that a certain variable proportion of the white cells of the blood thus treated exhibited no moving molecules, and apparently consisted solely of a nucleus and cell-wall. It is worthy of remark that this experiment amply demonstrates the inestimable advantages of high objectives (which some even yet pretend to doubt), for the remarkable movement of the contained molecules seems to have escaped the attention of Prof. Virchow, the great author of "The Cellular Pathology," (vide p. 181, Chance's translation, 1863). Although the observation was first made with the aid of a one 25th, yet afterwards knowing exactly what to look for, I had little difficulty in demonstrating to various gentlemen the revolving molecules thus brought into view with powers as low as the one 8th inch.

A portion of fetid pus from an abscess, and a specimen of mucus from the nasal fossa, under a like treatment gave similar results, which did not materially vary in numerous trials.

Tracing now the white blood-corpuscle from its condition of irregular outline, and amœboid movement, as observed in serum and in heavy urine, when the circumambient fluid approaches the density of 1028, through its rounded form with slightly more distinct nuclei, in the liquor puris, and in urine of lower specific gravity, we find that immersed in a rarer liquid, approximating to the mean density of the saliva (1005,) it has an accurately spherical outline, is more than twice the magnitude, and contains a number of minute actively moving molecules, thus exactly resembling in all sensible characters the true salivary corpuscle; and it therefore seems reasonably certain that the blood, under the appointed nervous influence, congesting the buccal mucous membrane and associated glands, moves slowly enough through its capillaries to allow some of its white globules to penetrate the walls of the vessels, as they are said to do those of the frog's mesentery in Cohnheim's experiment (Virchow, archiv; Band 40, 8, 38, u. s. w.), which under the influence of the rarer saliva, expanding them and setting them free to move their contained molecules constitute the bodies so long known to histologists as the corpuscles of the salivary fluid.—[*Balt. Med. Jour.*

